# Heregulin-HER3-HER2 signaling promotes matrix metalloproteinase-dependent blood-brain-barrier transendothelial migration of human breast cancer cell lines

**DOI:** 10.18632/oncotarget.2846

**Published:** 2015-02-19

**Authors:** Majid Momeny, Jodi M. Saunus, Flavia Marturana, Amy E. McCart Reed, Debra Black, Gianluca Sala, Stefano Iacobelli, Jane D. Holland, Dihua Yu, Leonard Da Silva, Peter T. Simpson, Kum Kum Khanna, Georgia Chenevix-Trench, Sunil R. Lakhani

**Affiliations:** ^1^ University of Queensland, UQ Center for Clinical Research, Herston, QLD, Australia; ^2^ QIMR Berghofer Medical Research Institute, Herston, QLD, Australia; ^3^ Mediapharma s.r.l., Chieti, Italy; ^4^ Department of Cancer Research, Max Delbruck Center for Molecular Medicine, Berlin, Germany; ^5^ Department of Molecular and Cellular Oncology, The University of Texas MD Anderson Cancer Center, Houston, TX, USA; ^6^ Pathology Queensland, The Royal Brisbane & Women's Hospital, Herston, QLD Australia; ^7^ The University of Queensland School of Medicine, Herston, QLD Australia

**Keywords:** Heregulin, HER2, HER3, blood-brain-barrier, matrix metalloproteinase, breast cancer-brain metastases

## Abstract

HER2-positive breast tumors are associated with a high risk of brain relapse. HER3 is thought to be an indispensible signaling substrate for HER2 (encoded by *ERBB2*) and is induced in breast cancer-brain metastases, though the molecular mechanisms by which this oncogenic dimer promotes the development of brain metastases are still elusive. We studied the effects of the HER3-HER2 ligand, heregulin (neuregulin-1, broadly expressed in the brain), on luminal breast cancer cell lines *in vitro*. Treatment of SKBr3 (*ERBB2*-amplified), MDA-MB-361 (*ERBB2*-amplified, metastatic brain tumor-derived) and MCF7 (HER2-positive, not *ERBB2*-amplified) cells with exogenous heregulin increased proliferation and adhesive potential, concomitant with induction of cyclin D1 and ICAM-1, and suppression of p27. All three cell lines invaded through matrigel toward a heregulin chemotactic signal in transwell experiments, associated with activation of extracellular cathepsin B and matrix metalloproteinase-9 (MMP-9). Moreover, heregulin induced breast cancer cell transmigration across a tight barrier of primary human brain microvascular endothelia. This was dependent on the activity of HER2, HER3 and MMPs, and was completely abrogated by combination HER2-HER3 blockade using Herceptin^®^ and the humanized HER3 monoclonal antibody, EV20. Collectively these data suggest mechanisms by which the HER3-HER2 dimer promotes development of metastatic tumors in the heregulin-rich brain microenvironment.

## INTRODUCTION

The development of brain metastases is a growing public health problem affecting more than 100,000 patients in the United States every year [1], including 10–30% of breast cancer patients [2, 3]. This complication is associated with severe morbidity and virtually 100% mortality, as currently there is no treatment strategy with proven efficacy. HER2-positive breast cancer patients are at particularly high risk [4, 5], with around half developing brain metastases during the course of disease [6]. HER2-targeted drug therapies delay the onset of brain metastases in these patients, and improve median survival after a diagnosis of metastatic brain relapse [7–10]. These observations indicate that HER2 plays a critical role in brain relapse. However, the molecular mechanisms underpinning this relationship have not been investigated in detail.

HER2 is an orphan member of the human epidermal growth factor receptor (HER/*ERBB*) family. It undergoes obligate heterodimerization with HER3, and to a lesser extent HER4 and EGFR [11–14]. The HER3-HER2 dimer is regarded as the major oncogenic unit in HER2-positive breast cancer [11, 15, 16]. Ligand-activated dimers transduce potent survival and proliferation signals through the PI3K-AKT and ERK1/2 pathways [11, 17]. HER3 has two ligands: Heregulin (HRG; also known as neuregulin-1 (*NRG1*)) and neuregulin-2. HRG is the better studied of the two and is broadly expressed in the brain by neurons, glia and the cerebral endothelium, functioning to promote survival, differentiation, migration and cytoprotection [18–20]. At least 17 HRG isoforms have been described, including secreted, membrane-bound and nuclear ‘back-signaling’ isoforms that are generated through a combination of alternate transcription start sites, splicing and post-translational processing [21]. Importantly, primary breast cancers that over-express HER3 are associated with a significantly higher rate of isolated brain metastases [22], and induction of HER3 is associated with development of brain metastases from both breast and lung cancers [23, 24]. Despite this, the functional relationships between HRG, HER3 and HER2 in breast cancer-brain metastases have not been elucidated.

HRG and HER2 signaling can also induce certain matrix metalloproteinase enzymes (MMPs) [25–28]. MMPs are zinc-dependent endopeptidases that degrade extracellular matrix (ECM) proteins. Their activities facilitate various normal physiologic processes (e.g. wound healing and organ development), and their dysregulation can be associated with pathological processes, including progression-associated changes in the tumor microenvironment. Regulation of MMP expression and activity is complex – they are expressed and stored as zymogens, secreted and activated on-demand by ‘convertases’ including other MMPs, and *in vivo*, their activities are fine-tuned according to the local balance between MMPs, TIMPs (tissue inhibitors of metalloproteinases) and other physiologic inhibitors [29] (e.g. the metastasis suppressor, RECK [30]). Therefore measurement of MMP expression is not a reliable surrogate for function. MMPs have been strongly implicated in the development of brain metastases from breast cancer [31–35]. For example, expression of MMP-9 is relatively higher in the brain-seeking MDA-MB-231 breast cancer cell line variant compared to parental and bone-homing counterparts [36], and ectopic expression of MMP-2 in MDA-MB-231 cells increased the incidence of brain metastases after intracardiac injection [37].

In order to establish distant brain metastases, disseminated breast cancer cells must initially traverse the blood-brain-barrier: a specialized endothelium that is resistant to diffusion of hydrophilic or large molecules by virtue of endothelial tight-junctions that are unique to the central nervous system. This specialized microvasculature is in close contact with astrocytic foot processes, pericytes and a thick basement membrane, which collectively facilitate the high substrate selectivity that is necessary to protect brain tissue from circulating pathogens and toxins, including chemotherapeutic agents [38, 39]. Extravasation of tumor cells across this barrier is therefore thought to be an active process. Following extravasation, tumor cells must establish growth-promoting interactions with the neural niche [40–43].

This study aimed to investigate molecular mechanisms by which HER3-HER2 signaling may promote the development of brain metastases from breast cancer. In light of the inferred associations between HER3, HER2, MMP activity and brain metastases, and the ubiquitous expression of heregulin in the brain, we hypothesized that heregulin may activate molecular mechanisms conducive to the establishment and growth of HER2-positive breast cancer cells in the brain microenvironment.

## RESULTS

### Expression of *ERBB3* and *NRG1* isoforms in breast cancer cell lines

**Figure 1:**
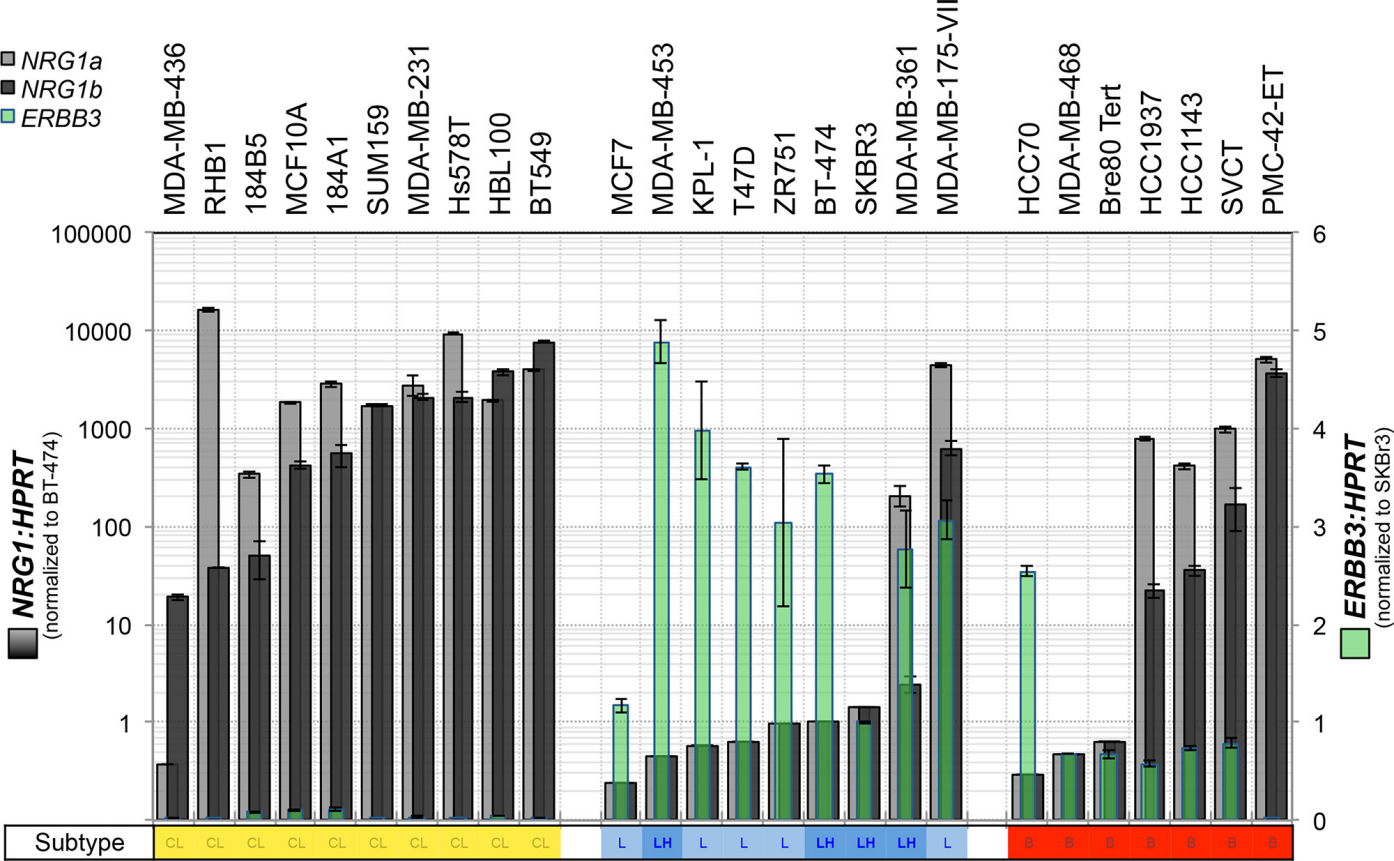
Expression of heregulin and HER3 in claudin-low, luminal and basal A breast cancer cell lines The breast cancer cell lines indicated were cultured to sub-confluence then total RNA was isolated for quantitative RT-PCR analysis of heregulin (neuregulin-1 α and β splice isoforms: *NRG1α* and *NRG1β*) and *ERBB3*. Data shown are means ± standard deviation. We observed an inverse association between *ERBB3* and *NRG1* with reciprocal expression in luminal compared to claudin-low (basal B) breast cancer cell lines.

To characterize the expression of heregulin (*NRG1* gene) and HER3 (*ERBB3* gene) in breast cancer cell line models, and select the most appropriate lines for functional experiments, we investigated the relative baseline mRNA expression levels of *ERBB3* and *NRG1* (α and β heregulin splice isoforms) in a large panel of breast cancer cell lines by quantitative reverse transcription-PCR (qRT-PCR). The gene expression profile-based molecular subtypes of these cell lines (luminal, luminal-*ERBB2*-amplified, basal-A and basal-B/claudin-low) were derived from published reports [44–46], marked in Figure 1. This screening experiment revealed an inverse association between *ERBB3* and *NRG1* expression, with highest levels of *ERBB3* in luminal cell lines, and highest levels of *NRG1* in claudin-low cell lines, consistent with their mesenchymal-like phenotype [46] (Figure 1). *ERBB3/NRG1* expression phenotypes were mixed in basal-A cell lines.

### Paracrine activation of HER2-HER3 in luminal breast cancer cell lines

We next investigated the responses of three representative *ERBB3*-expressing cell lines to treatment with exogenous heregulin (HRG): MDA-MB-361, MCF7 and SKBr3. These three cell lines are luminal-like when stratified based on transcriptomic profile [44–46]. Other key features to note are that MDA-MB-361 and SKBr3 harbor *ERBB2* amplification, MDA-MB-361 was isolated from a breast cancer-derived metastatic brain tumor, and SKBr3 cells do not express estrogen receptor (ER-negative) [44]. All three lines are capable of colonizing the brain in animal models ([47, 48] and unpublished observations).

**Figure 2:**
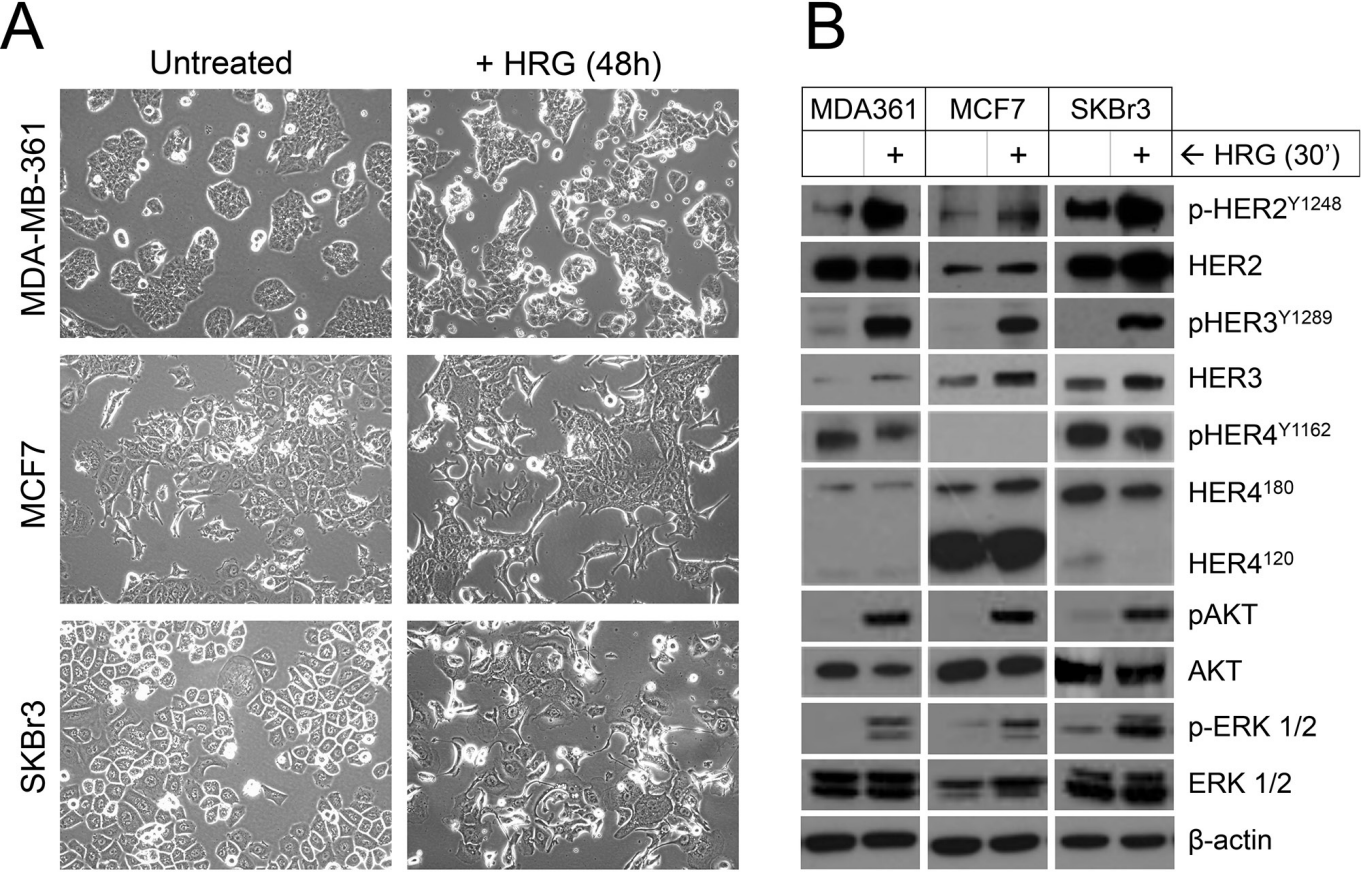
Treatment of luminal HER2+ breast cancer cell lines with exogenous HRG alters cell morphology and activates signaling through HER2, HER3, AKT and ERK **(A)** Serum-starved cells were treated with HRG for 48 h then imaged by light microscopy (images acquired at 20x magnification). **(B)** Serum-starved cells were treated with HRG for 30 min then total and phosphorylated HER, AKT and ERK isoforms were quantified by Western blot. β-actin was used as the loading control.

To begin to examine the effects of exogenous HRG, cells were deprived of serum (‘serum-starved’) before HRG treatment, since serum contains many growth factors including HRG itself. Forty-eight hours of HRG treatment resulted in noticeable morphological changes, including stellate features and pseudopodia formation by MCF7 and SKBr3 cells (Figure 2A), consistent with other reports suggesting HRG treatment induces an epithelial-to-mesenchymal phenotypic shift in these cell lines [49, 50]. Morphologic change for MDA-MB-361 was consistent with the other two cell lines but more subtle overall, with cells becoming less cohesive and developing some stellate projections.

We also investigated HER3-HER2 downstream signaling 30 min after HRG treatment. All three cell lines responded to exogenous HRG with phosphorylation of HER3 and its preferred dimerization partner HER2, but not the other HRG receptor HER4 (Figure 2B). There was also HRG-induced phosphorylation of AKT and ERK1/2, important downstream targets of HER2 that regulate tumor cell survival, proliferation and invasion [17]. Though of lesser magnitude than the phosphorylation induction, there was also an increase in total HER3 protein levels. The short time frame of this experiment suggests this may involve post-transcriptional mechanisms, such as protein stabilization or translation efficiency.

In contrast to the HER2/HER3-positive luminal cell lines, three representative claudin-low cell lines (Hs578T, MDA-MB-231 and SUM-159-PT; Figure 1) did not show induction of HER3 expression or phosphorylation following treatment with exogenous HRG (Supplementary Figure 1).

### Exogenous HRG treatment induces cell line-dependent proliferation and adhesion of luminal breast cancer cells *in vitro*

**Figure 3:**
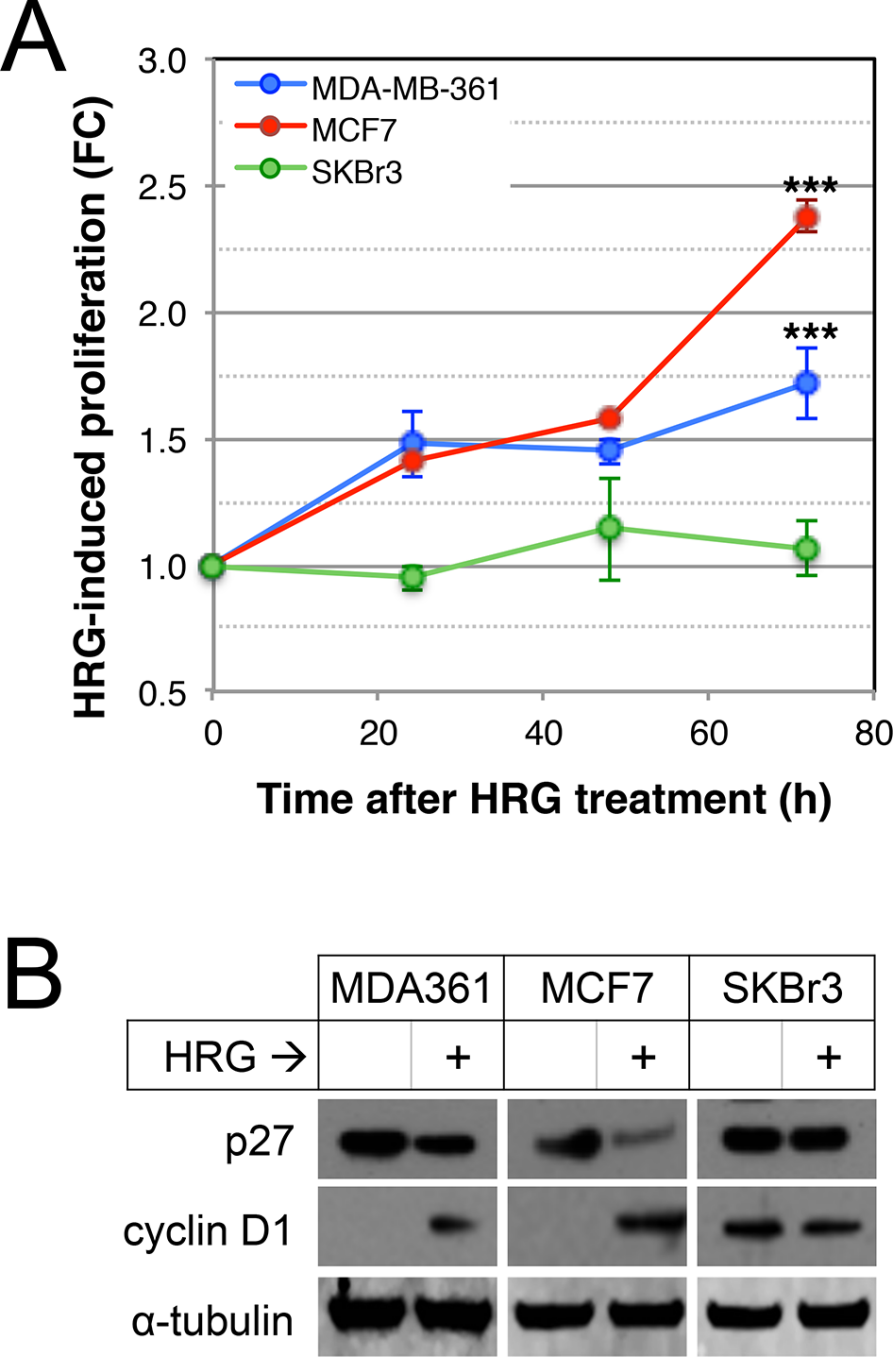
Effects of HRG on proliferation of luminal HER2+ breast cancer cell lines **(A)** Serum-starved cells were treated with HRG and proliferation rate was assessed using an MTT assay over the following 72 h. Data shown are means ± standard deviation, normalised to an untreated control group for each cell line at each timepoint. ***p < 0.0001 according to unpaired, 2-tailed student's *t*-tests comparing treated vs untreated cells at 72 hours post-treatment. FC, fold-change. **(B)** Serum-starved cells were treated with HRG for 48 h, then expression of p27 and cyclin D1 proteins was quantified by Western blot (loading control: α-tubulin).

Since AKT and ERK1/2 were potently activated by HRG in three luminal breast cancer cell lines and they are known to induce tumor cell proliferation in other contexts [51], we investigated the effects of HRG on proliferation of HRG-treated versus untreated MDA-MB-361, MCF7 and SKBr3 cells. As shown in Figure 3A, HRG induced a time-dependent proliferative response in MDA-MB-361 and MCF7, but not SKBr3 cells. Others have demonstrated that HER2 promotes proliferation through deregulation of cell cycle checkpoints [52]. We therefore investigated the effects of HRG on cyclin D1 and p27 protein levels by Western blot analysis. As shown in Figure 3B, HRG treatment attenuated p27 and induced cyclin D1 expression in the two proliferative cell lines.

**Figure 4:**
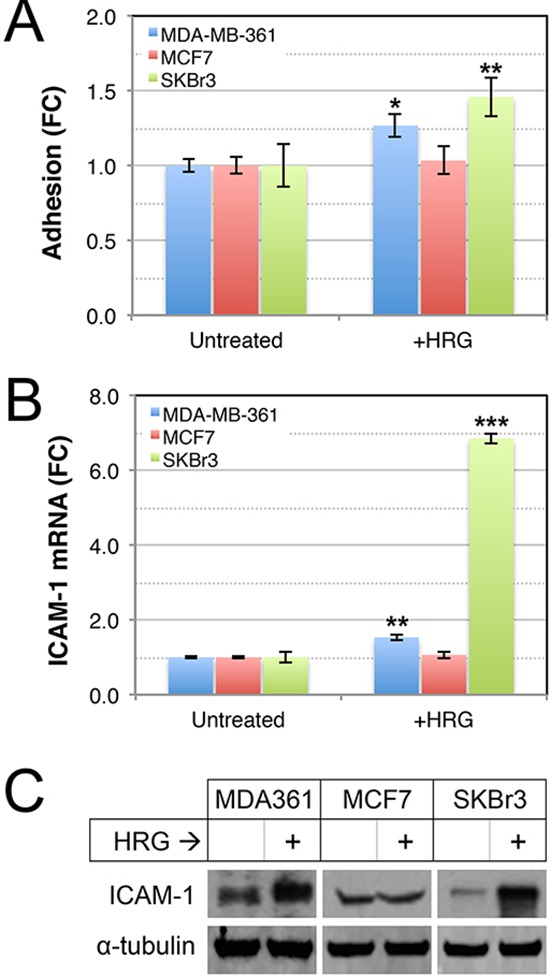
Effects of HRG on adhesive abilities of luminal HER2+ breast cancer cell lines **(A)** HRG increases adhesion of MDA-MB-361 and SKBr3 cells to collagen-I. Serum-starved cells were treated with HRG for 48 h, allowed to adhere to collagen-I-coated dishes for 15 min then adhesion potential was determined using optical density measurements of treated versus untreated controls. *p = 0.05–0.01, **p = 0.01–0.001 (2-tailed, unpaired student's *t*-tests). HRG increases expression of ICAM-1 RNA **(B)** and protein **(C)** in MDA-MB-361 and SKBr3 cells. Cells were treated as above, then total RNA or protein were prepared for quantitative real-time PCR or Western blot of ICAM-1 expression respectively (*HPRT1* or α-tubulin loading controls, respectively). **p = 0.001–0.0001; ***p < 0.0001 (2-tailed, unpaired student's *t*-tests). FC, fold-change.

Tumor cell adhesion to extracellular matrix proteins enhances survival and metastatic potential of circulating tumor cells [53], and adhesion of circulating tumor cells to the brain endothelium is thought to be a critical step preceding endothelial retraction and active extravasation [54, 55]. To investigate whether HER2-HER3 signaling increases the adhesive properties of luminal breast cancer cell lines, we assayed adhesion of HRG-treated cells to collagen I, which is a substrate for a range of cell adhesion molecules. HRG enhanced the adhesion of MDA-MB-361 and SKBr3 cells to collagen I (Figure 4A), concomitant with induction of ICAM-1 (Figure 4B–4C), a β2-integrin receptor associated with enhanced invasion, motility and metastasis in breast cancer [56–58]. Collectively these data show that exogenous HRG promotes proliferation and adhesion of luminal breast cancer cell lines, though these responses could be context-dependent since they were not consistent across the cell lines tested.

### Exogenous HRG induces luminal breast cancer cell line invasion and secreted protease activity

**Figure 5:**
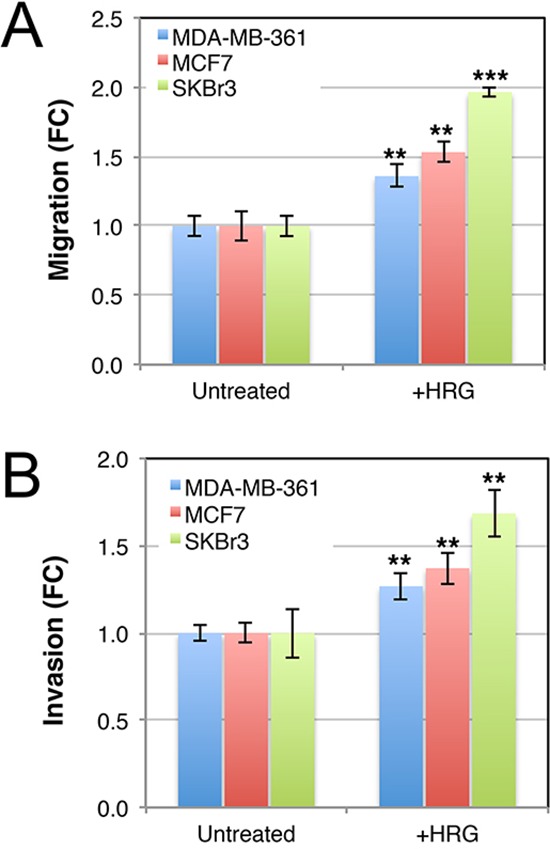
Luminal HER2+ breast cancer cell lines invade across matrigel toward a heregulin chemotactic signal Serum-starved cells were seeded in 8 μm transwell chambers **(A)** or 8 μm transwell chambers in which the membranes were pre-coated with matrigel **(B)**. HRG was supplemented in the lower chamber, and media was changed regularly to maintain a concentration gradient. After 48 h, cells on the lower surfaces of the porous membranes were quantified by crystal violet staining. Data shown are means ± standard deviation, normalised to the untreated control group for each cell line. **p = 0.05–0.01, **p = 0.01–0.001 (unpaired, 2-tailed student's *t*-tests of HRG-treated cells compared to untreated controls). FC, fold-change.

AKT signaling induces aggressive breast cancer cell behavior and others have reported that HRG induces motility and invasion through ECM proteins (e.g. [50, 59–61]). Consistent with this, transwell assay experiments showed that the three cell lines migrated toward a HRG chemotactic signal (Figure 5A). Moreover, this response was maintained after coating the transwell inserts with matrigel (Figure 5B). These data show that HRG promotes both migratory and invasive behavior of luminal breast cancer cell lines, which otherwise migrate very poorly *in vitro*.

**Figure 6:**
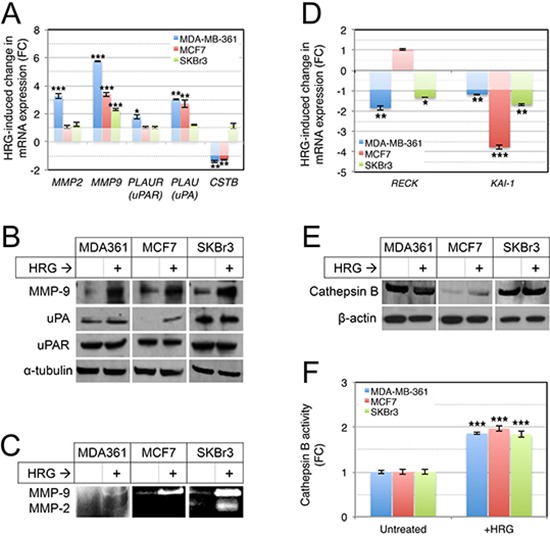
Treatment of luminal breast cancer cell lines with exogenous HRG increases extracellular protease activity **(A, B)** HRG increases expression of proteolytic cascade proteins. Serum-starved cells were treated with HRG for 48 h, then total RNA or protein were isolated from the cells for qRT-PCR and Western blot analyses respectively (*HPRT1* and α-tubulin were used as normalization and loading controls, respectively). **(C)** HRG increases secreted MMP-2 and MMP-9 proteolytic activities. Starved cells were treated with HRG as above and then conditioned media was concentrated and analysed for MMP-2 and MMP-9 activity by gelatin zymography (enzymatic activity is proportional to the intensity of the white bands). **(D)** HRG represses expression of *RECK* and *KAI1* metastasis suppressor genes. qRT-PCR analysis was performed as for (A). HRG treatment does not substantially alter cathepsin B protein expression **(E)**, but increases extracellular cathepsin B proteolytic activity **(F)**. Cathepsin B expression was analyzed by Western blot analysis as for (B), with β-actin as the loading control. Enzyme activity was assayed using a fluorometric enzyme activity assay. *p = 0.01–0.001, **p = 0.001–0.0001, ***p < 0.0001 (unpaired, 2-tailed student's *t*-tests comparing treated to untreated control samples). FC, fold-change.

Since this invasive activity requires remodeling of ECM proteins, and AKT-associated invasion is marked by secretion of several proteases that are important in invasion and metastasis [62], we next investigated expression of MMP-2, MMP-9, urokinase plasminogen activator receptor (*PLAU*; uPAR) and its ligand (*PLAUR*; uPA), and cathepsin B (*CSTB*) in the HRG-treated cells. Using qRT-PCR analysis we found HRG induced cell line-dependent increases in expression of *MMP2*, *PLAUR* and *PLAU*, and a modest but significant decrease in expression of *CSTB* (Figure 6A). *MMP9* was consistently induced in all three cell lines (Figure 6A). This was also evident at the protein level, with Western blot analysis confirming induction of MMP-9 protein in all three cell lines, and variable changes for the other proteolytic proteins (Figure 6B).

Ultimately we were interested in common HRG-induced changes in the secretion and extracellular activity of ECM proteases, and so we assessed the proteolytic activities of MMP-9 and MMP-2 activity in conditioned media from the HRG-treated cells by gelatin zymography (gelatin is a substrate for both enzymes). This experiment confirmed that HRG-mediated induction of MMP-9 was associated with activation of extracellular MMP-9 activity in cultures of all three cell lines, though the total amount was relatively lower in MDA-MB-361 compared to SKBr3 and MCF7 cells (Figure 6C). MMP-2 activity was induced in MDA-MB-361 and SKBr3 cells.

Others have previously reported that in both breast tumors and cell lines, there is a negative association between the expression of MMP-9 and one of its natural inhibitors, RECK [30], a key breast cancer metastasis suppressor gene. Furthermore, *RECK* is repressed in brain metastases compared to primary breast cancers [63]. The *KAI1* (CD82) metastasis suppressor has also been implicated in MMP-9 repression [64]. We therefore investigated expression of *KAI1* and *RECK* by qRT-PCR and found that HRG treatment repressed both genes (Figure 6D), suggesting this could be one mechanism by which HRG increases extracellular MMP-9 activity. Interestingly, the two *RECK*-suppressed cell lines (MDA-MB-361 and SKBr3) are *ERBB2*-amplified, and RECK is known to functionally oppose oncogenic HER2 signaling by interfering with HER3 dimerization [65].

Finally, we investigated the expression and activity of extracellular cathepsin B, as this has been implicated in mediating invasive behavior of HER2-positive breast cancer cells [66], and activation of MMP-9 and infiltrative tumor cell growth in glioma [67–69]. There was no substantial change in cathepsin B expression following treatment with exogenous HRG (Figure 6E), but there was a significant increase in extracellular cathepsin B activity in all three breast cancer cell lines (Figure 6F). Others have shown that PI3K mediates cathepsin B secretion by lysosomal exocytosis [70], and therefore increased secretion of cathepsin B may be one mechanism by which HRG increases its extracellular activity in breast cancer cell lines.

### Exogenous HRG induces transmigration of breast cancer cell lines across a tight barrier of primary human brain microvascular endothelial cells

**Figure 7:**
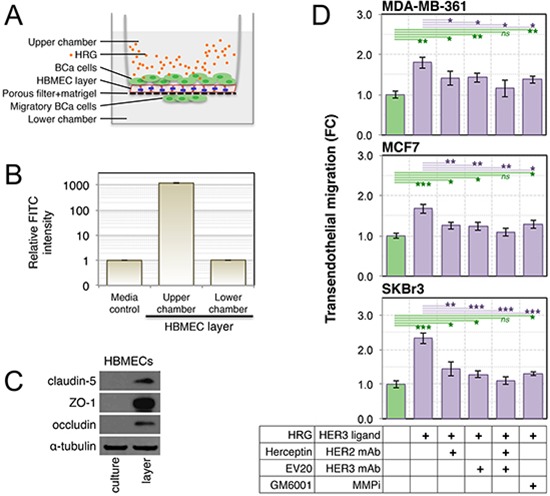
Treatment of luminal breast cancer cell lines with exogenous HRG increases their endothelial transmigration activity **(A)** Schematic of the *in vitro* blood-brain-barrier model used to assay transendothelial migration of HRG-treated breast cancer cell lines (blue lines = tight junctions). Primary human brain microvascular endothelial cells (HBMECs) were seeded into matrigel-coated transwell chambers, then allowed to form a confluent layer. Serum-starved breast cancer cell lines were then seeded over the layer). **(B)** The functional integrity of the HBMEC layer was validated by measuring dextran-FITC (40 kDa) flux from the upper to lower transwell chambers. **(C)** HBMEC expression of tight junction proteins in layer-forming culture conditions was confirmed by Western blot analysis. **(D)** Breast cancer cell line transendothelial migration activity was measured in response to HRG ligand with and without drug treatments as indicated. Data shown are means ± standard deviation (*n* = 3 from a representative experiment). The statistical significance of differences between treatments and the untreated control was determined using unpaired, 2-tailed student's *t*-tests (*p = 0.05–0.01, **p = 0.01–0.001 and ***p < 0.001). BCa, breast cancer; FC, fold-change; mAb, monoclonal antibody; MMPi, matrix metalloproteinase inhibitor.

MMP-2 and MMP-9 have been associated with degradation of endothelial tight junction proteins, permeabilization of the blood-brain-barrier (BBB) and subsequent brain colonization in mouse models of leukemia [71]. Therefore we investigated whether active MMP isoforms in conditioned media from HRG-treated breast cancer cell lines are sufficient to stimulate transmigration across an endothelial barrier. We established an *in vitro* model of the BBB using primary human brain microvascular endothelial cells (HBMECs) and matrigel to simulate the brain endothelium and basement membrane respectively (Figure 7A). The integrity of this barrier was validated by measuring dextran-FITC diffusion (Figure 7B), and by confirming strong induction of tight junction proteins claudin-5, ZO-1 and occludin in the HBMEC layer (Figure 7C) as described [72].

Treatment of co-cultured breast cancer cell lines with exogenous HRG (Figure 7A) caused active migration of all three cell lines across the tight HBMEC layer (Figure 7D). This response was attenuated upon inhibition of HER2, HER3 or MMP activity, assessed by supplementing the upper chambers with saturating doses of humanized monoclonal antibodies against HER2 and HER3 (Herceptin^®^ and EV20 respectively) or the broad-spectrum MMP inhibitor, GM6001. Treatment with GM6001 did not completely abrogate the response, indicating that MMPs may not be the only transmigration mechanism activated by HRG-HER3-HER2 signaling. It is noteworthy that complete inhibition of transmigratory activity was only achieved through combined blockade of HER2 and HER3 (Herceptin^®^ + EV20; Figure 7D, green lines).

## DISCUSSION

The development of brain metastases is a devastating complication that affects 10–30% of women with breast cancer [2], causing challenging neurological symptoms including headaches, cognitive impairment and seizures [73]. Current treatments can prolong life expectancy and improve quality-of-life, though ultimately they are not curative. Molecular targeted drug therapy is critically under-utilized in the clinic, partly because of deficiencies in our understanding of the molecular mechanisms involved in the seeding and subsequent proliferation of disseminated cells in the brain. Research in this area is now beginning to illuminate some of the mechanisms by which tumor cells exploit and remodel the local microenvironment to facilitate metastatic outgrowth [40–42, 74–76].

Heregulin is critical for the normal development and function of the nervous system. It is expressed by neurons, glia and brain microvascular endothelia, where its functions include promoting glial cell survival and differentiation, neural precursor cell differentiation and migration, and endothelial cell protection from oxidative injury [18–20]. Consistent with the idea that HRG is a brain growth factor exploited by metastatic cells, HER3 is induced and activated in brain metastases compared to matching breast and lung cancers [23, 24] and patients with HER2-positive breast cancer are at high risk of brain relapse [4, 6]. Moreover, primary breast cancers over-expressing HER3 are more likely to relapse as isolated brain metastases than non-HER3-overexpressing tumors [22].

In this study, we found that exposure to HRG stimulated the transendothelial migration of HER2/3-expressing breast cancer cell lines across a tight barrier of primary human brain microvascular endothelia and an associated matrigel layer, and that this was at least partly mediated by MMPs (Figure 7). Specifically, exposure to HRG increased the extracellular activity of MMP-9 in three cell lines, and MMP-2 in two of these lines (Figure 6). Other studies have implicated MMP-2 and -9 in the development of brain metastases [31–34], and the current study now suggests that this could be at least partly due to enhancing vascular permeability. Since HRG is expressed by brain microvascular endothelia [20], these data raise the possibility that HRG-HER3-HER2 signaling is involved in extravasation from the brain microvasculature *in vivo*, particularly since we also found that HRG increases breast cancer cell adhesion potential (Figure 4). Indeed, MMPs 2 and 9 can mediate vascular leakage in experimental models of cerebral ischemic injury by degrading endothelial tight junction complexes [77–79]. HRG-mediated vascular permeabilization could also be important in established metastases with increasing metabolic demands.

Adjuvant Herceptin^®^ therapy for HER2-positive breast cancer delays the onset of brain metastases [10], and this latency is further extended by the HER2 dimerization blocker Perjeta^®^ [8]. In this context, it is noteworthy that Herceptin^®^ and the humanized HER3 antibody EV20 [80] conferred additive suppression of transmigration in our blood-brain-barrier experiments (Figure 7). There are likely to be multiple mechanisms enabling endothelial transmigration and consequent establishment of micrometastases *in vivo* (for example, we also found that exposure to exogenous HRG reduces expression of *KAI1* and *RECK* metastasis suppressor genes), however these *in vitro* experiments may provide some molecular insight into the aforementioned clinical observations.

The cell lines used in this study all migrated and invaded through extracellular matrix proteins towards an HRG chemotactic signal (Figure 5), concomitant with increased activity of extracellular proteases (Figure 6). Consistent with other reports [60, 81], we observed cell proliferation in response to HRG exposure (Figure 3). Collectively these data suggest that HRG-HER3-HER2 signaling could be involved in several aspects of brain metastasis development. *In vivo* experiments modeling these steps with inhibition of HER2-HER3 dimer function are required in the future.

This study has potential implications in translational oncology, and warrants further investigation into the possibility of targeting the HRG-HER3-HER2 axis for management of brain metastases from HER2-positive breast cancer.

## MATERIALS AND METHODS

### Breast cancer cell lines and HRG activation assay

The breast cancer cell lines used in this study were obtained from the American Type Culture Collection (ATCC). The panel of cell lines included three molecular subtypes previously defined by expression array profiling and unsupervised cluster analysis and/or surrogate immunohistochemical markers [44–46]: claudin-low (basal B): MDA-MB-436, MDA-MB-231, RHB1, 184B5, MCF10A, 184A1, SUM159, Hs578T, HBL100 and BT549; basal-A: HCC70, MDA-MB-468, Bre80Tert, HCC1937, HCC1143, SVCT, PMC-42-ET; luminal: MCF7, KPL-1, T-47D, ZR751, MDA-MB-175-VII); and luminal/ERBB2-amplified: MDA-MB-453, BT-474, SKBr3 and MDA-MB-361. MDA-MB-361 is the only commercially available breast cancer cell line that was derived from a metastatic brain tumor. All cell lines were authenticated by STR profiling (Cell ID™ system; Promega) and were routinely checked for mycoplasma infection (MycoAlert™; Lonza). Cell cultures were maintained at 37°C in 5% CO_2_ in a humidified incubator and cultured according to ATCC recommendations.

For heregulin (HRG) activation experiments, cells were routinely seeded at predetermined densities in regular culture medium, then switched from the recommended amount of fetal bovine serum (FBS) to 0.1% FBS (serum-starved conditions) for 24 hours. Cultures were then supplemented with 50 ng/mL HRG-β1 (Sigma) and cultured for either 30 min (signaling analysis) or 48 h (functional experiments including assaying expression of downstream targets of HER2-HER3 signaling).

### Analysis of gene expression by quantitative real time reverse transcription-PCR (qRT-PCR)

Trizol (Invitrogen) was used to isolate total RNA from cultured cells. cDNA was prepared using 1 μg of RNA from each sample with the PrimeScript RT reagent kit (Takara Bio Inc.). qPCR was then performed in triplicate on a StepOnePlus instrument using SYBR green (Applied Biosystems) according to standard procedures (see Table 1 for PCR primers). Melt curve analysis was performed to verify amplification of single PCR products. Hypoxanthine phosphoribosyl transferase1 (*HPRT1*) was amplified as normalizer and fold change in expression of each target mRNA relative to *HPRT1* was calculated according to the 2^−ΔΔct^ relative expression formula [82]. HER3 qPCR was performed using the *ERBB3* TaqMan® expression assay (Hs00951455_m1; Applied Biosystems).

**Table 1:** Primers used for qRT-PCR

Gene	Accession no.	Forward primer (5′-3′)	Reverse primer (5′-3′)
*NRG1α*	NM_013964	AAACCAAGAAAAGGCGGAGGAGCT	GAGGGCGATGCAGATGCCGG
*NRG1β*	NM_013956	GCCAGCTTCTACAAGCATCTTGGGA	GGAGGGCGATGCAGATGCCG
*HPRT1*	NM_000194	TGGACAGGACTGAACGTCTTG	CCAGCAGGTCAGCAAAGAATTTA
*MMP2*	NM_004530	CTTCCAAGTCTGGAGCGATGT	TACCGTCAAAGGGGTATCCAT
*MMP9*	NM_004994	GGGACGCAGACATCGTCATC	TCGTCATCGTCGAAATGGGC
*PLAU* (uPA)	NM_002658	TCAAAAACCTGCTATGAGGGGA	GGGCATGGTACGTTTGCTG
*PLAUR* (uPAR)	NM_002659	TGTAAGACCAACGGGGATTGC	AGCCAGTCCGATAGCTCAGG
*KAI1*	NM_002231	GCCGACAAGAGCAGTTTCATC	AGGAAAGCAAAGTACAGCCCC
*RECK*	NM_021111	TGTGAACTGGCTATTGCCTTG	GCATAACTGCAACAAACCGAG
*ICAM1*	NM_000201	TTGGGCATAGAGACCCCGTT	GCACATTGCTCAGTTCATACACC
*CSTB*	NM_147780	CTGTCGGATGAGCTGGTCAAC	TCGGTAAACATAACTCTCTGGGG

### Western blot analysis

Total protein extracts were prepared in RIPA buffer (50 mM Tris-HCl, pH 8.0, 150 mM NaCl, 1.0% NP-40, 0.5% sodium deoxycholate and 0.1% SDS) containing fresh protease and phosphatase inhibitors (Thermo Scientific) for 30 min at 4°C. Thirty to fifty μg of lysate was resolved by SDS-PAGE, transferred to PVDF membrane (Immobilin P, Millipore) then probed with primary antibodies (Table 2) followed by horseradish peroxidase (HRP)-conjugated secondary antibodies (Sigma). Two different HER3 antibodies were used to confirm the increase in HER3 protein levels 30 min after HRG treatment. Blots were probed with α-tubulin and β-actin as loading controls.

**Table 2:** Primary antibodies used for western blot analysis

Antigen	Clone	Supplier
HER3	2F12	Millipore
HER3	C-17	Santa Cruz
HER2	Polyclonal	Millipore
pHER2^Y1248^	Polyclonal	Cell Signaling Technology
pHER3^Y1289^	21D3	Cell Signaling Technology
Akt	Polyclonal	Cell Signaling Technology
pAkt^S473^	D9E	Cell Signaling Technology
ERK1/2	137F5	Cell Signaling Technology
p-ERK1/2^T202/Y204^	197G2	Cell Signaling Technology
Cathepsin B	G60	Cell Signaling Technology
Cyclin D1	H-295	Santa Cruz Biotechnology
ICAM-1	H-108	Santa Cruz Biotechnology
p27	C-19	Santa Cruz Biotechnology
MMP-9	Polyclonal	Sigma
uPA	H-140	Sigma
uPAR	Polyclonal	Sigma
ZO-1	ZO1-1A12	Invitrogen
Occludin	OC-F10	Invitrogen
α-tubulin	4G1	Abcam
β-actin	8H10D10	Cell Signaling Technology

### Cell proliferation assay

A microculture tetrazolium test (MTT) was performed to determine cell proliferation after treatment of the cells with recombinant HRG. Briefly, cells were plated onto 96-well plates at a density of 4×10^4^/well for 24 h and then starved in 0.1% FBS for 24 h. The cells were then treated with 50 ng/mL of HRG for 24, 48 and 72 h. Untreated cells were used as the control group. 100 μL of MTT (0.5 mg/ml) (Sigma) was added to each well and the cultures were further incubated at 37°C for 2 h. After dissolving the precipitated formazan with 100 μL of dimethyl sulfoxide (DMSO), the optical density was measured at 570 nm.

### Cell adhesion assay

Adhesion experiments were conducted as described [14]. Cells were seeded into 6-well plates and after 24 h, washed three times with PBS and starved in 0.1% FBS overnight. The starved cells were treated with 50 ng/mL HRG for 48 h and seeded on collagen I coated 60 mm dishes (Biocoat Cell Environments; Becton Dickinson). After 15 min, cells were washed with cold PBS, stained with 0.5% crystal violet, lysed with 30% acetic acid and the optical density was measured at 590 nm.

### Gelatin zymography

Gelatin zymography was carried out as described [83]. Briefly, conditioned media from HRG-treated and untreated cells was collected and centrifuged at high speed for 10 min to pellet cell debris. Protein from the conditioned media was then concentrated as appropriate using a Vacufuge® plus (Eppendorf). Five μg of secreted protein were applied to 10% polyacrylamide gels copolymerized with 1 mg/mL gelatin (Sigma). After electrophoresis, gels were rinsed in 2.5% Triton X-100 (3×30 min) to remove SDS, followed by incubation at 37°C overnight in incubation buffer (0.15 M NaCl, 10 mM CaCl_2_, 0.02% NaN_3_ in 50 mM Tris-HCl, pH 7.5). Gels were then stained (0.5% Coomassie Brilliant Blue) and destained with 7% methanol and 5% acetic acid. Areas of enzymatic activity appeared as clear bands over the dark background.

### Cell migration and invasion assays

Cell migration was assayed in 24-well, 6.5-mm-internal-diameter transwell plates (8.0 μm pore size; Costar Corp.). Serum-starved cells were placed in the upper chambers, and the lower chamber was filled with media containing 0.1% FBS (control), with or without HRG supplementation (50 ng/mL). Media in both chambers was changed every 6 hours to maintain an HRG gradient. Cells were allowed to migrate for 48 h. After this time, cells on the upper surfaces of the filters were removed by wiping with a cotton swab and migrated cells on the undersides of the filters were fixed with methanol, stained with crystal violet, lysed with 30% acetic acid and the optical density was measured at 590 nm. For invasion, experiments were essentially conducted as above, except that transwell filters were pre-coated with matrigel (1:10 dilution in media; BD Biosciences).

### Cathepsin B activity assay

To investigate the effect of HRG on the activity of secreted cathepsin B, conditioned media from HRG-treated and untreated cells was centrifuged at 10,000 rpm for 15 min to remove cell debris, then concentrated appropriately using the Vacufuge® plus (Eppendorf). We used a fluorometric cathepsin B activity assay (Abcam) according to the manufacturer's instructions.

### Blood-brain-barrier transendothelial migration assay

Primary human brain microvascular endothelial cells (HBMECs) and HBMEC culture reagents were purchased from Cell Systems and cells were routinely cultured in CSC Complete Medium on fibronectin (Invitrogen) pre-coated surfaces, according to the manufacturer's recommendations. Two×10^4^ HBMECs were seeded on matrigel-coated 0.4 μm transwell filters (Costar Corp.) in 200 μL of CSC complete medium. The lower compartment was filled with 600 μL of the same medium. Cells were grown for 4 d to allow complete formation of HBMEC tight junctions (TJs). The impermeability of this *in vitro* BBB model to small solutes was assayed by measuring dextran-FITC (Invitrogen) flux from the top to bottom chambers. The media was changed to 1% FBS-CSC media plus CultureBoost (Cell Systems) for 24 h. Dextran-FITC (1 mg/mL; Invitrogen) was added to the upper chamber and after 20 min, 50 μL media from upper and lower chambers was removed and fluorescence was measured using a Paradigm™ Detection Platform (Beckman Coulter; 485 nm excitation, 520 nm emission).

For transmigration assays, 2×10^4^ HBMEC cells were seeded into matrigel-coated (BD Biosciences) 24-well transwell inserts (8 μm pores; Costar Corp.). The cells were maintained for 4 d to allow the TJ formation, then media from upper and lower chambers was changed to 1% FBS-CSC media plus CultureBoost for 24 h (as above). Serum-starved breast cancer cell lines (1×10^5^ for MCF7 and SKBr3, or 2×10^5^ for MDA-MB-361) were seeded into the upper chamber in 100 μL of their regular media containing 0.1% FBS, then allowed to attach for 2 h. Cultures were then treated with HRG, (50 ng/mL; Sigma), GM6001 (20 μg/mL; Calbiochem), Herceptin^®^ (20 μg/mL; Experimental Pharmacology Oncology, Berlin) and/or EV20 (20 μg/mL; Mediapharma) for 48 h. The upper surfaces of the transwell filters were then gently wiped clean with a cotton swab to remove non-migrating cells and cells on the undersides were fixed with methanol, stained with crystal violet, lysed with 30% acetic acid and the optical density was measured at 590 nm

### Statistical analysis

Data are expressed as mean ± standard deviation (SD). All experiments were performed in triplicate. For statistical analysis, unpaired, two-tailed *t*-tests were applied. *P* values of less than 0.05 were considered significant.

## SUPPLEMENTARY FIGURE


