# Supplementary material for: Heregulin-HER3-HER2 signaling promotes matrix metalloproteinase-dependent blood-brain-barrier transendothelial migration of human breast cancer cell lines
Source: Oncotarget. 2015 Feb 19;6(6):3932–46. doi: 10.18632/oncotarget.2846 (PMC4414164; doi:10.18632/oncotarget.2846)
Supplement: Supplementary file 1 [file oncotarget-06-3932-s001.pdf]

SUPPLEMENTARY FIGURE

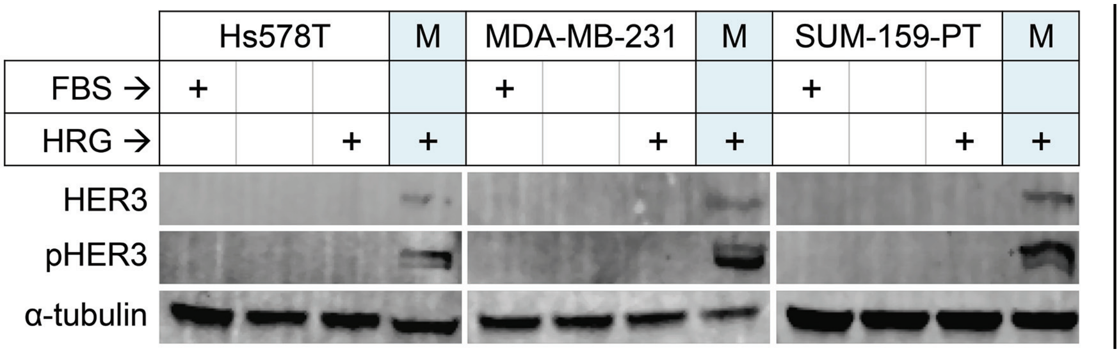

Supplementary Figure 1: Treatment of claudin-low breast cancer cell lines with exogenous HRG does not alter HER3 protein expression or activation. Consistent with qRT-PCR data (Figure 1), claudin-low cell lines did not express HER3 or pHER3 under regular culture conditions (‘FBS’=fetal bovine serum). Treatment with HRG for 30 min did not induce expression of HER3 or pHER3. Serum-starved MCF7 cells treated with HRG for 30 min were included as positive controls (M; Fig. 2).
